# Supplementary material for: Proteome-wide solubility and thermal stability profiling reveals distinct regulatory roles for ATP
Source: Nat Commun. 2019 Mar 11;10:1155. doi: 10.1038/s41467-019-09107-y (PMC6411743; doi:10.1038/s41467-019-09107-y)
Supplement: Supplementary file 1 — Supplementary Information [file 41467_2019_9107_MOESM1_ESM.pdf]

Proteome-wide solubility and thermal stability profiling reveals distinct regulatory roles for ATP

Sridharan et al

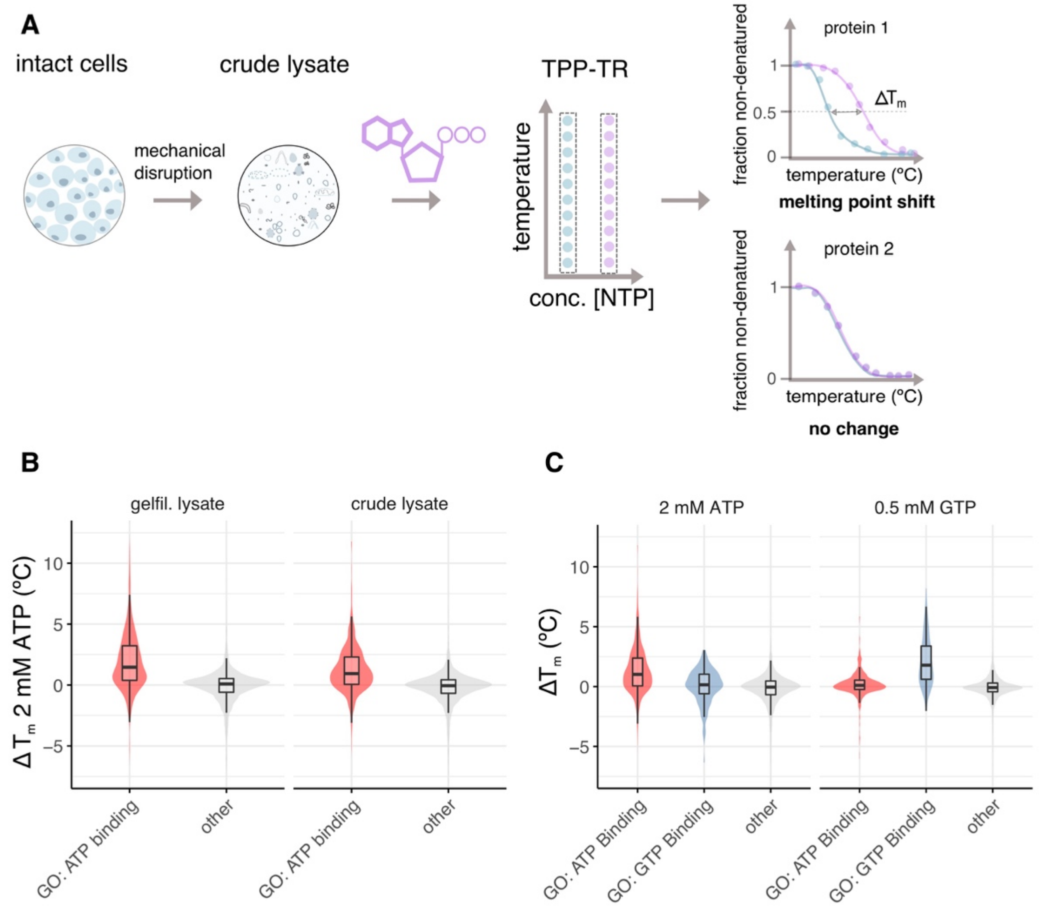

Supplementary Figure 1. (A) Experimental setup of TPP on crude lysates. Dotted rectangular box corresponds to one TMT10 experiment. (**B**) Boxplot comparing melting point shifts induced by 2mM ATP in crude and gel filtered lysate from two independent experiments. (**C**) Boxplot comparing of melting point shifts induced by 2mM ATP and 0.5 mM GTP in crude lysate from two independent experiments. Violin plots represent relative densities. Center line in box plots is the median, the bounds of the boxes are the 75 and 25% percentiles i.e., the interquartile range (IQR) and the whiskers correspond to the highest or lowest respective value or if the lowest or highest value is an outlier (greater than 1.5 * IQR from the bounds of the boxes) it is exactly 1.5 * IQR. Source data are provided for figure panels B and C as a Source Data file.

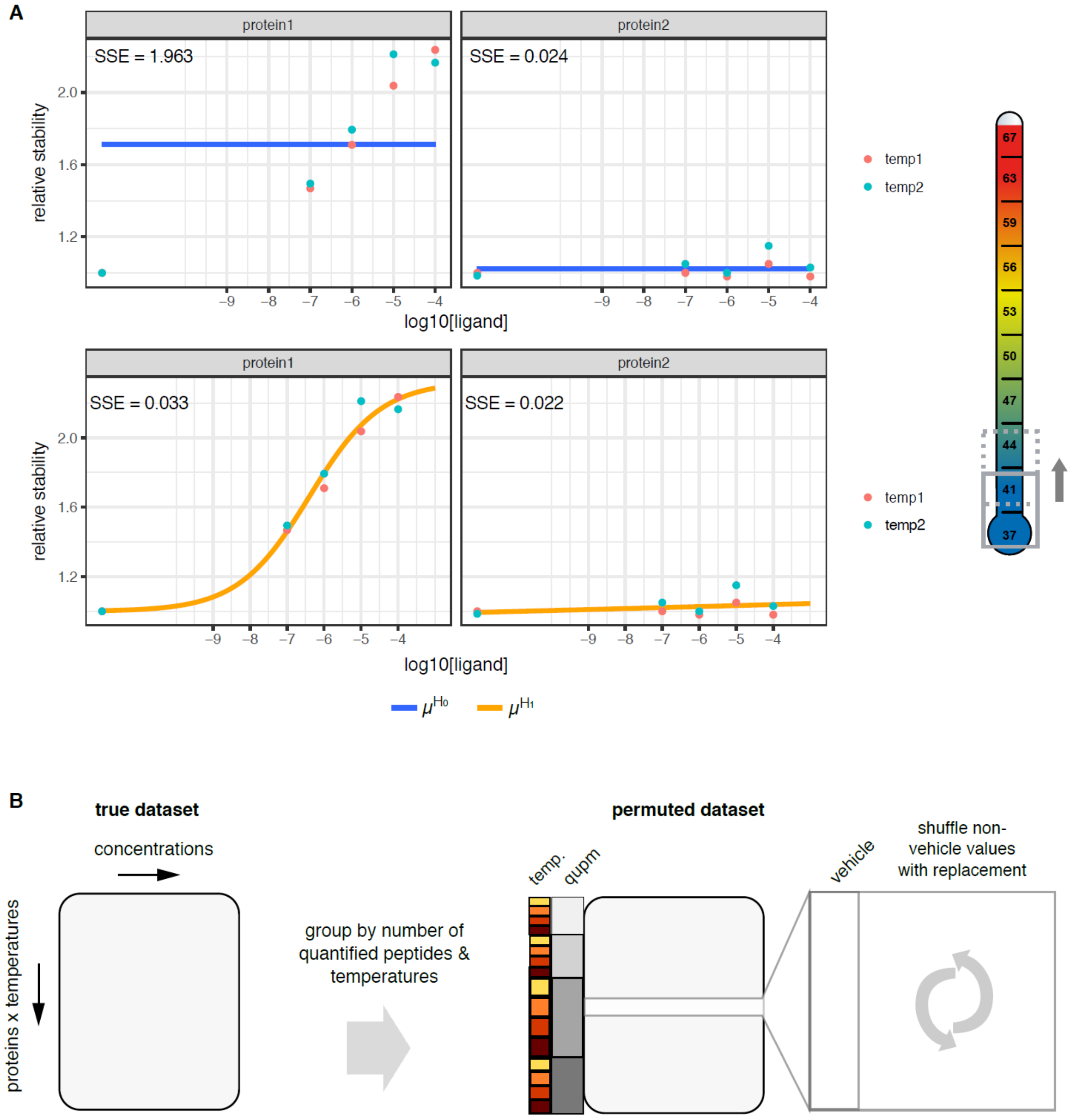

Supplementary Figure 2. (A) Schematic illustration of the sliding temperature window approach to determine drug dosage-dependent protein stabilization of individual proteins. Relative stability values of two exemplary proteins (protein1 and protein2) are shown for different drug concentrations (including vehicle) at two adjacent temperatures. Whereas protein1 shows a much smaller sum of squared error (SSE) when fitted by the parametric dose-response model in comparison with the H0 model. This is not the case for protein2, therefore protein1 will achieve a high and protein2 a low F-statistic. **(B)** Illustration of the recursive permutation of the data to estimate FDR. The true dataset (proteins x temperatures times concentrations) is grouped by temperature and number of peptides used for quantification. Within these groups relative stability values are permuted with replacement across proteins and concentrations, leaving out the reference values of the vehicle condition.

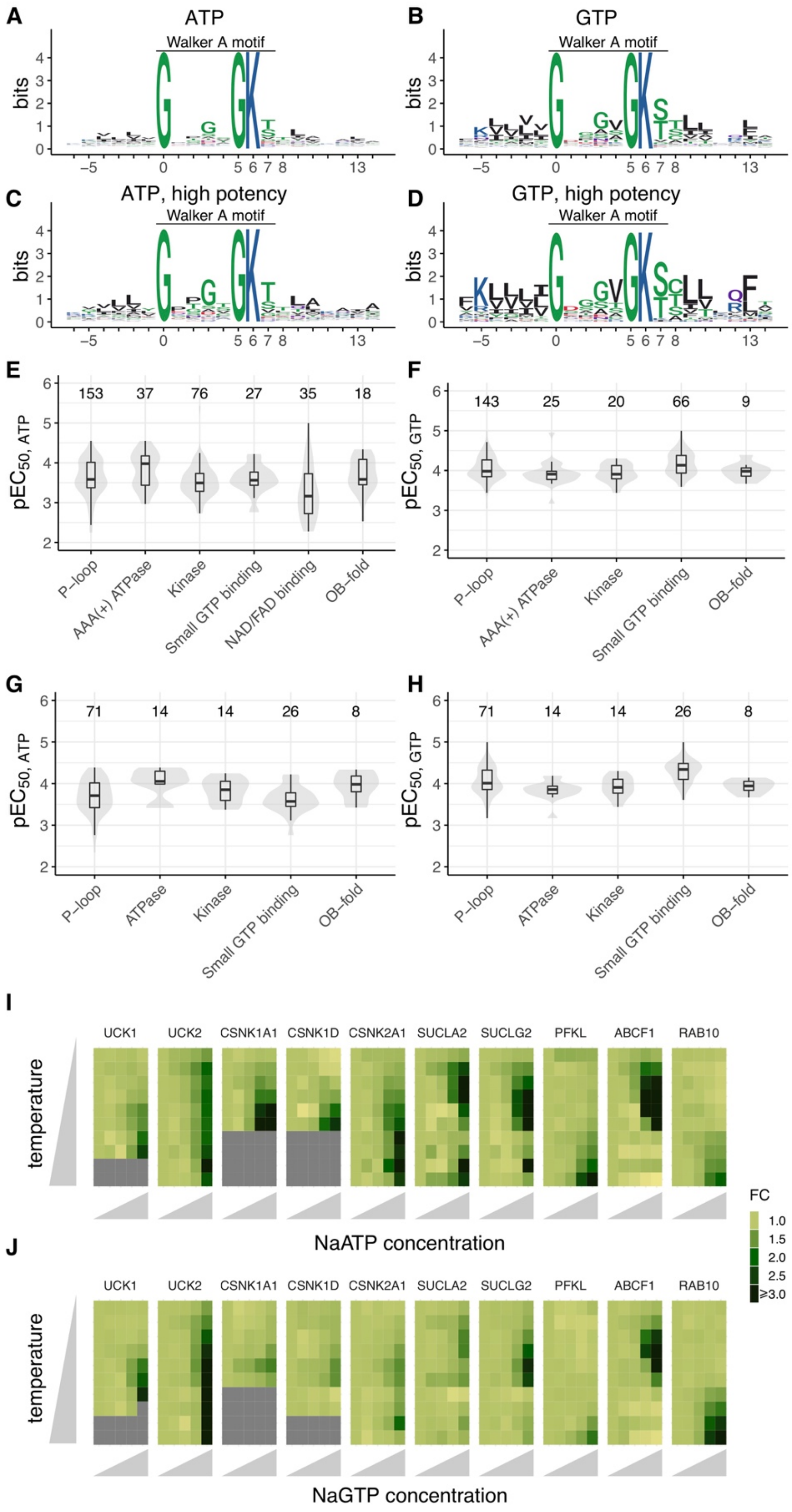
A
ATP
Walker A motif
bits
B
GTP
C
ATP, high potency
D
GTP, high potency
E
153
37
76
27
35
18
pEC50, ATP
P–loop
AAA(+) ATPase
Kinase
Small GTP binding
NAD/FAD binding
OB–fold
F
143
25
20
66
9
pEC50, GTP
G
71
14
14
26
8
ATPase
H
I
UCK1
UCK2
CSNK1A1
CSNK1D
CSNK2A1
SUCLA2
SUCLG2
PFKL
ABCF1
RAB10
temperature
NaATP concentration
FC
1.0
1.5
2.0
2.5
≥3.0
J
NaGTP concentration

Supplementary Figure 3. **(A-B)** Sequence motif analysis of residues flanking the Walker-A motif of proteins that were stabilized by ATP or GTP. **(C-D)** Sequence motif analysis of residues flanking the Walker-A motif of proteins that were potently (pEC_{50} > (mean (pEC_{50}) + 1 standard deviation)) stabilized by ATP and GTP. **(E-F)** Distributions of pEC_{50} values of all proteins stabilized by GTP and ATP partitioned according to different domains. **(G-H)** Distributions of pEC_{50} values of proteins stabilized by both GTP and ATP partitioned according to different domains. **(I-J)** Representative heat maps showing relative fold changes in protein abundances upon treatment with ATP (0.005, 0.05, 0.5 and 2 mM) or GTP (0.001, 0.01, 0.1 and 0.5 mM) compared to untreated crude lysate (first column on each plot) with increasing temperature (y-axis: 42.0, 44.1, 46.2, 48.1, 50.4, 51.9, 54.0, 56.1, 58.2 and 60.1 ˚C). Dark gray fields correspond to missing values. Violin plots represent relative densities. Center line in box plots is the median, the bounds of the boxes are the 75 and 25% percentiles i.e., the interquartile range (IQR) and the whiskers correspond to the highest or lowest respective value or if the lowest or highest value is an outlier (greater than 1.5 * IQR from the bounds of the boxes) it is exactly 1.5 * IQR. Numbers above violin plots represent number of proteins. Source data are provided for all figure panels as a Source Data file.

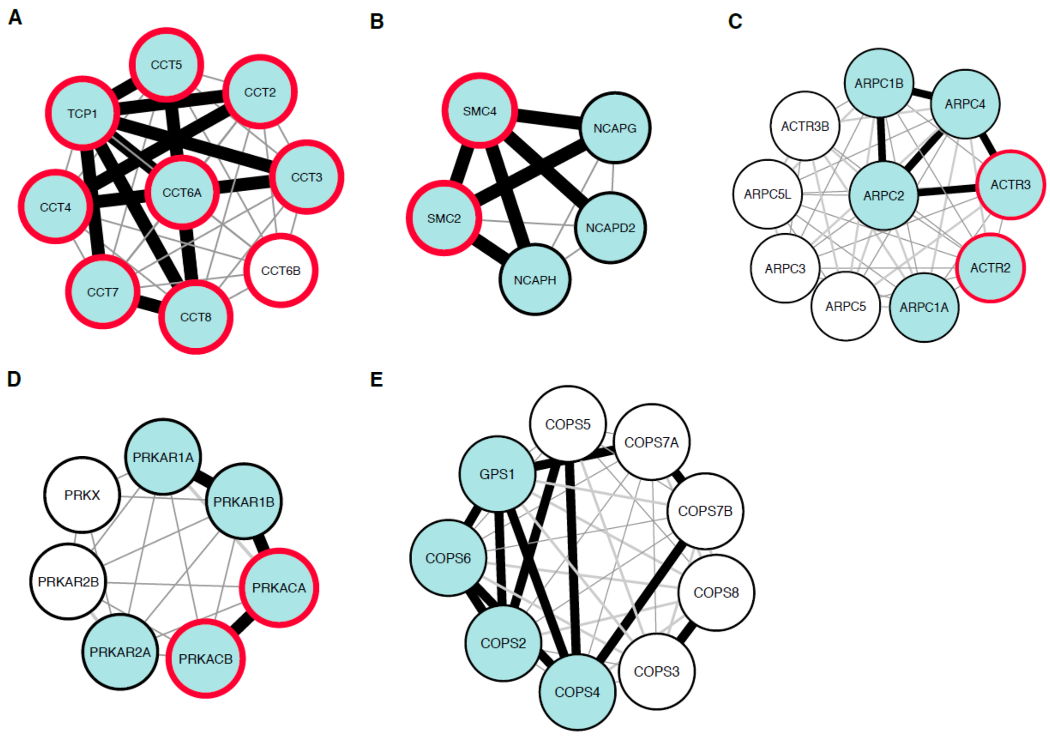

Supplementary Figure 4. Selected examples of protein complexes. **(A)** Chaperonin containing-T complexes, **(B)** Condensin, **(C)** Arp2/3 complex, **(D)** Protein-kinase-A complex and **(E)** COP9 signalosome that stabilize upon ATP addition. Nodes represented as filled circles are proteins that stabilize upon ATP addition and the nodes with red outline are known ATP binding proteins. Thick edges between proteins represent proteins that exhibit similar melting behavior in TPP dataset, with a Euclidean distance between the between curves lesser than 0.02. Source data are provided for all figure panels as a Source Data file.

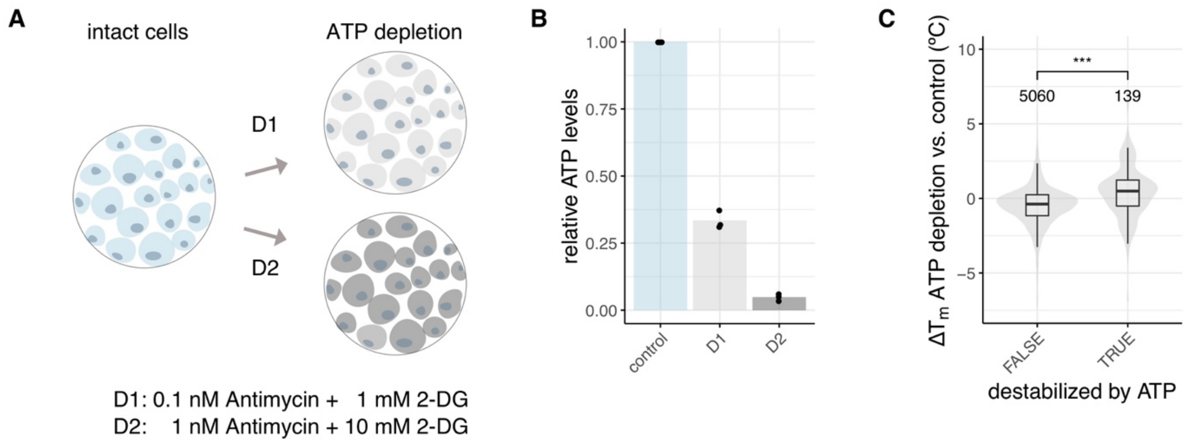

Supplementary Figure 5. (A) Illustration of inhibitor concentrations used to deplete cellular ATP levels **(B)** Quantification of relative ATP levels after treatment with doses D1 and D2 for inhibitors using Cell-titer Glo assay from three independent experiments. **(C)** Changes in melting points in ATP depleted compared to vehicle conditions of proteins that were destabilized in crude lysate experiments upon addition of ATP from three independent experiments. Significance levels obtained from a Wilcoxon signed-rank test were encoded as *p < 0.05, **p < 0.01, and ***p < 0.001. Violin plots represent relative densities. Center line in box plots is the median, the bounds of the boxes are the 75 and 25% percentiles i.e., the interquartile range (IQR) and the whiskers correspond to the highest or lowest respective value or if the lowest or highest value is an outlier (greater than 1.5 * IQR from the bounds of the boxes) it is exactly 1.5 * IQR. Numbers above violin plots represent number of proteins. Source data are provided for figure panels B and C as a Source Data file.

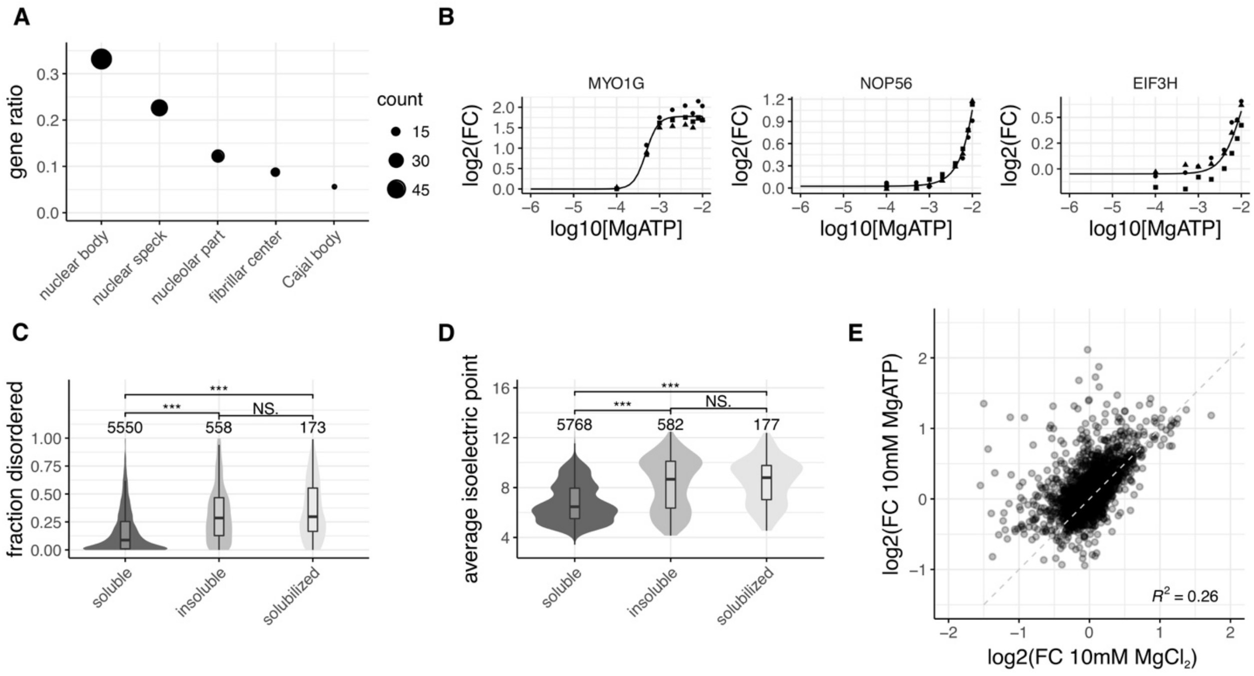

Supplementary Figure 6. (A) Dotplot of gene ontology cellular compartment enrichment results featuring membrane-less organelle terms for comparison of proteins solubilized by ATP with all identified proteins. **(B)** Solubility profiles of MYO1G, NOP56, and EIF3H, following ATP treatment, y-axis represents the $\log_2$ ratio of NP40 processed ATP and vehicle treated conditions. **(C-D)** Distributions of fractions of disordered regions and isoelectric points of proteins part of the soluble, insoluble, and ATP solubilized proteomes. Significance levels obtained from a Wilcoxon signed-rank test were encoded as $*p < 0.05$, $**p < 0.01$, and $***p < 0.001$. Violin plots represent relative densities. Center line in box plots is the median, the bounds of the boxes are the 75 and 25% percentiles i.e., the interquartile range (IQR) and the whiskers correspond to the highest or lowest respective value or if the lowest or highest value is an outlier (greater than 1.5 * IQR from the bounds of the boxes) it is exactly 1.5 * IQR. Numbers above violin plots represent number of proteins. Source data are provided for all figure panels as a Source Data file.

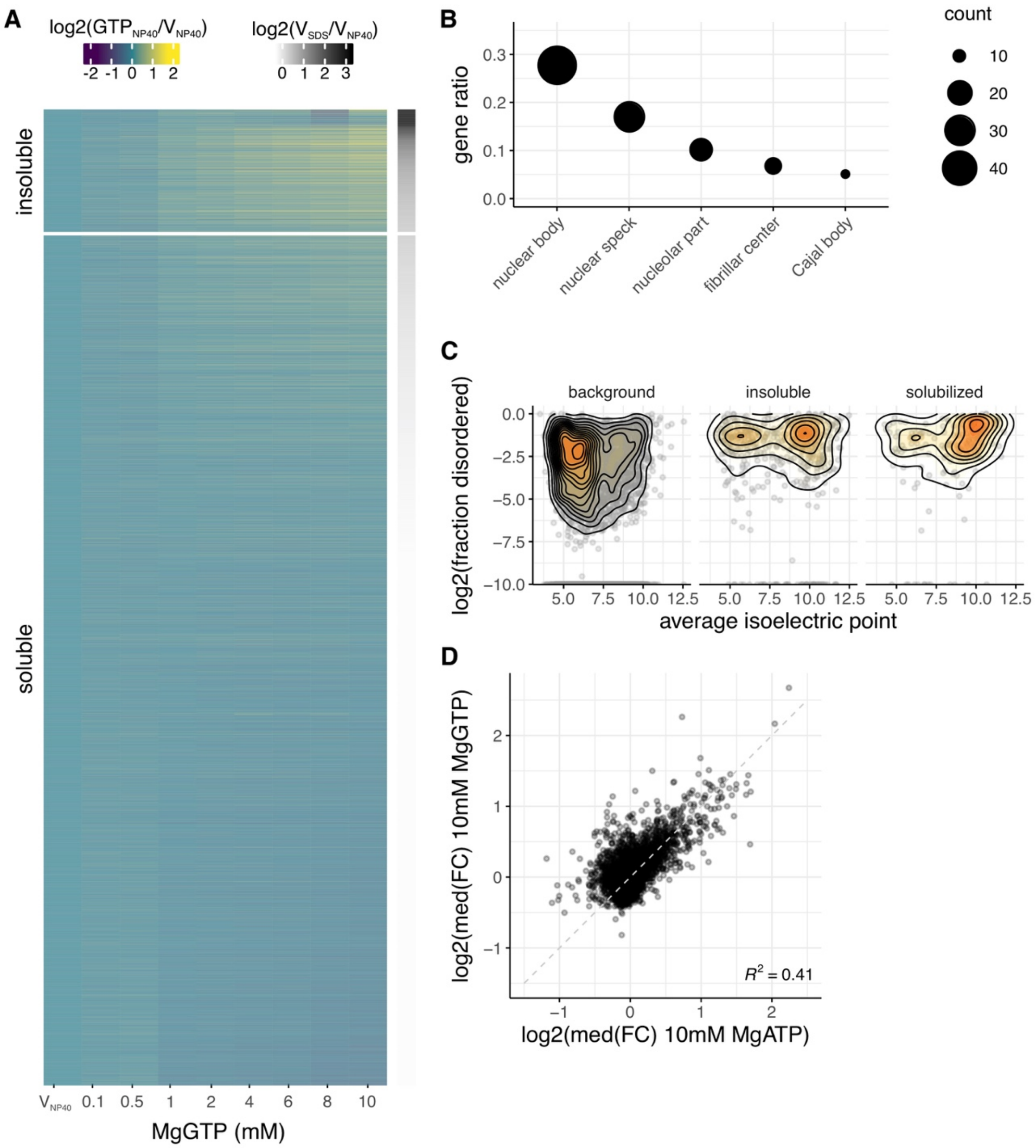

Supplementary Figure 7. (A) Heat map representation of proteome solubility. The grey scale represents the $\log_2$ ratio between NP40 and SDS processed vehicle condition. The color scale represents the $\log_2$ ratio between NP40 processed GTP treated and vehicle treated conditions. **(B)** Dotplot of gene ontology cellular compartment enrichment results featuring membrane-less organelle terms for comparison of proteins solubilized by GTP with all identified proteins. **(C)** 2D density plot of $\log_2$ fraction disorder vs. average isoelectric points of soluble and insoluble

proteins, and of those solubilized by GTP. **(D)** Scatter plot comparing solubility changes of proteins induced by 10 mM ATP and 10 Mm GTP. Source data are provided for all figure panels as a Source Data file.

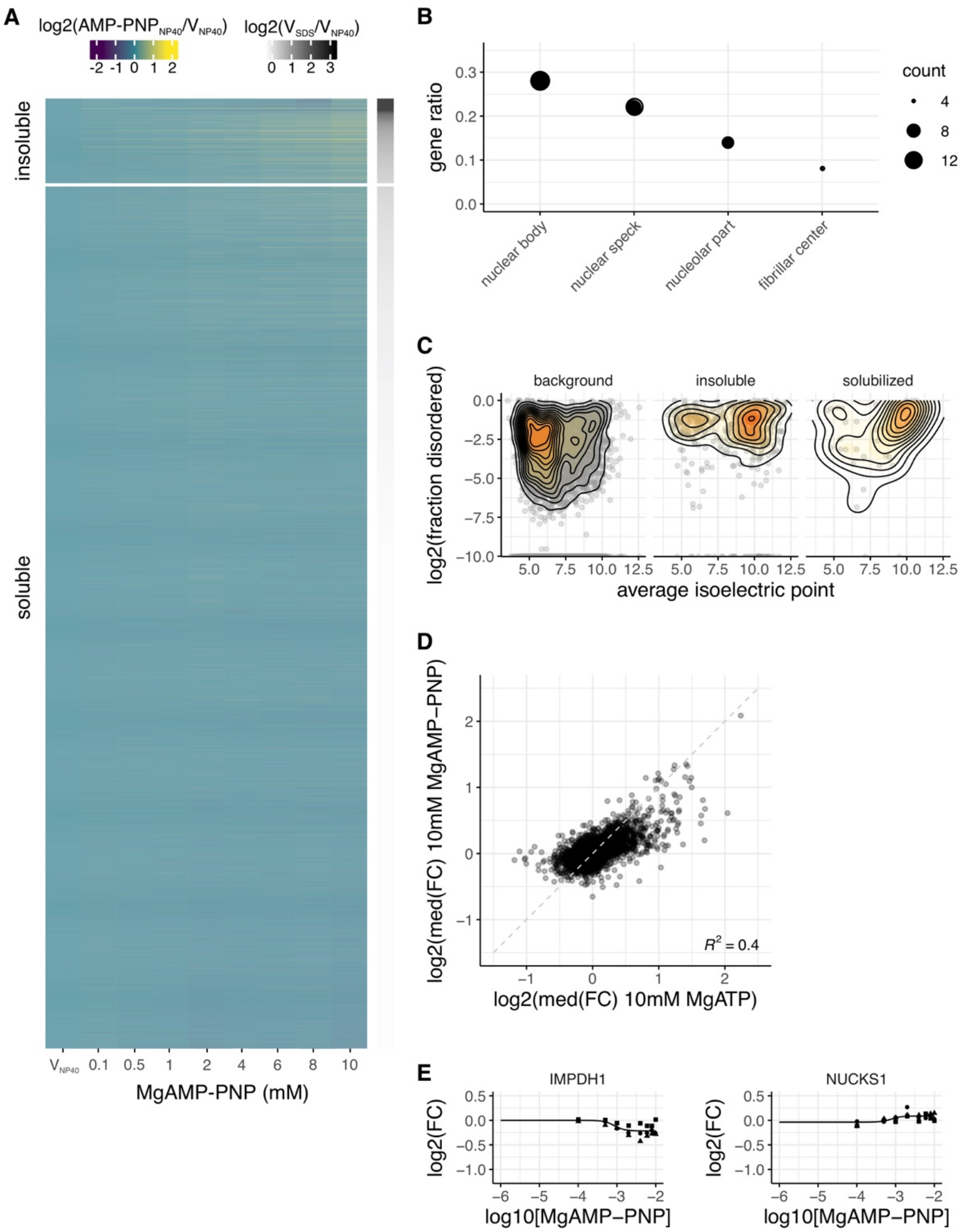

Supplementary Figure 8. **(A)** Heat map representation of proteome solubility. The grey scale represents the log_2 ratio between NP40 and SDS processed vehicle condition. The color scale represents the log_2 ratio between NP40 processed AMP-PNP treated and vehicle treated conditions. **(B)** Dotplot of gene ontology cellular compartment enrichment results featuring membrane-less organelle terms for comparison of proteins solubilized by AMP-PNP with all identified proteins. **(C)** 2D density plot of log_2 fraction disorder vs. average isoelectric points of soluble and insoluble proteins, and of those solubilized by AMP-PNP. **(D)** Scatter plot comparing solubility changes of proteins induced by 10 mM ATP and 10 Mm AMP-PNP. (**E**) Solubility profile

for IMPDH1 and NUCKS1, fold change (FC) in y-axis represents the ratio of NP40 processed AMP-PNP treated and vehicle treated conditions. Source data are provided for all figure panels as a Source Data file.

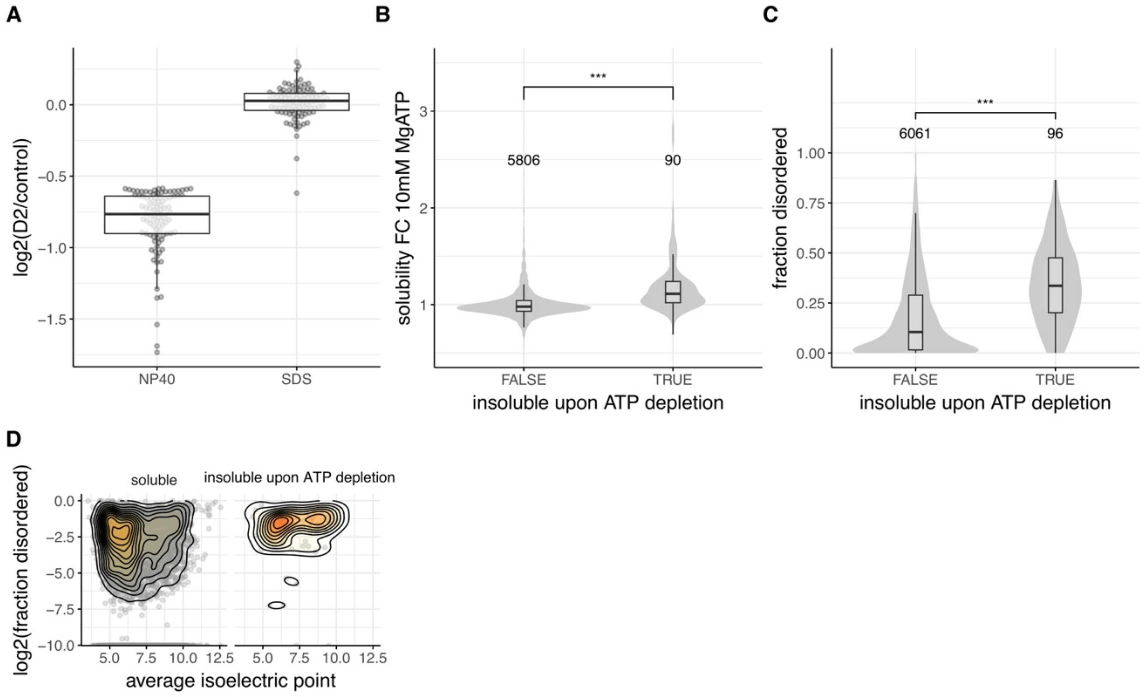

Supplementary Figure 9. **(A)** Distribution of $\log_2$ ratios of ATP depleted and control conditions processed with NP40 (left) or SDS (right) of the 107 proteins that decreased in solubility upon ATP depletion. **(B)** distribution of $\log_2$ ratios of 10 mM ATP treated and vehicle conditions processed with NP40 in the crude lysate experiment of the proteins that decreased in solubility upon ATP depletion (94 out 107 were identified in the crude lysate experiment). Significance levels obtained from a Wilcoxon signed-rank test were encoded as *p < 0.05, **p < 0.01, and ***p < 0.001. **(C)** Distributions of fractions of disordered regions of proteins that decreased in solubility upon ATP depletion. Significance levels obtained from a Wilcoxon signed-rank test were encoded as *p < 0.05, **p < 0.01, and ***p < 0.001 **(D)** 2D density plot of $\log_2$ fraction disorder vs. average isoelectric points of soluble and insoluble proteins upon ATP depletion. Violin plots represent relative densities. Center line in box plots is the median, the bounds of the boxes are the 75 and 25% percentiles i.e., the interquartile range (IQR) and the whiskers correspond to the highest or lowest respective value or if the lowest or highest value is an outlier (greater than 1.5 * IQR from the bounds of the boxes) it is exactly 1.5 * IQR. Numbers above violin plots represent number of proteins. Source data are provided for all figure panels as a Source Data file.

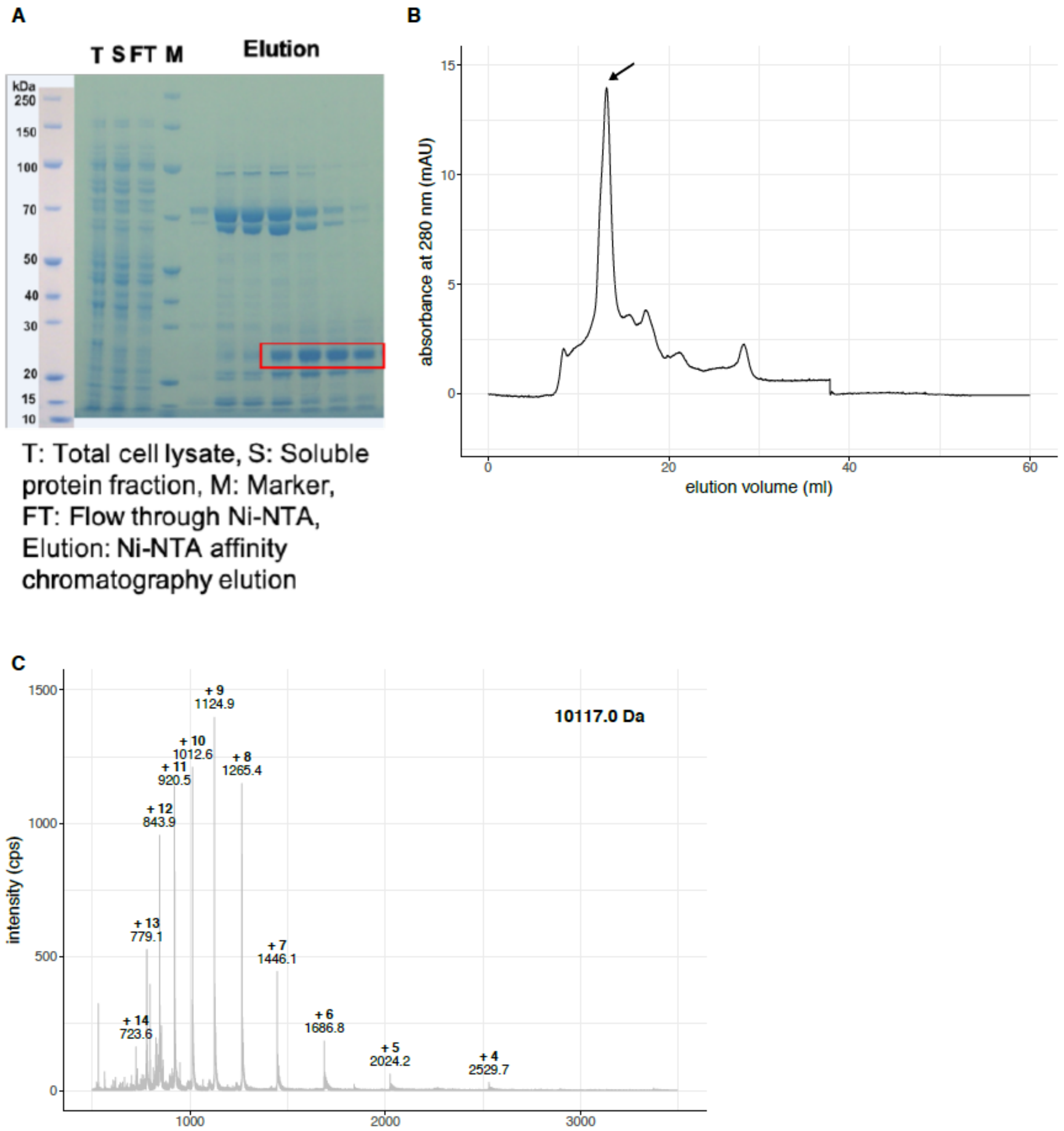

Supplementary Figure 10. **(A)** SDS-PAGE showing the Ni-NTA purification of recombinant BANF1 expressed as a fusion protein with N-terminal His-Thioredoxin tag with a TEV cleavage site (red box). **(B)** Gel filtration chromatogram of recombinant BANF1 cleaved from its tag (arrow indicates the elution of BANF1) using Superdex 75 column. BANF1 is a 10 KDa protein which eluted at 13.5 ml, indicating that it exists as dimer in solution. **(C)** Mass spectrum of BANF1 acquired on a Q-TOF mass spectrometer. Deconvolution of the spectra resulted in 10117.0 Da which matches the calculated mass of recombinant BANF1(10116.9 Da). Source data are provided for figure panels B and C as a Source Data file.
