# Supplementary material for: Proteome-wide solubility and thermal stability profiling reveals distinct regulatory roles for ATP
Source: Nat Commun. 2019 Mar 11;10:1155. doi: 10.1038/s41467-019-09107-y (PMC6411743; doi:10.1038/s41467-019-09107-y)
Supplement: Supplementary file 3 — Description of Additional Supplementary Files [file 41467_2019_9107_MOESM3_ESM.docx]

**Description of Additional Supplementary Files**

**Supplementary Data 1.** Thermal proteome profiling of crude and gel filtered lysate treated with 2 mM Na-ATP

**Supplementary Data 2.** Thermal proteome profiling of crude lysate treated with 2 mM ATP and 0.5 mM GTP.

**Supplementary Data 3.** 2D-thermal proteome profiling in the presence of ATP and GTP.

**Supplementary Data 4.** Thermal proteome profiling after ATP depletion.

**Supplementary Data 5.** Solubility proteome profiling in the presence of ATP.

**Supplementary Data 6.** Solubility proteome profiling in the presence of GTP.

**Supplementary Data 7.** Solubility proteome profiling in the presence of AMP-PNP.

**Supplementary Data 8.** Thermal proteome profiling in the presence of 10 mM Mg-ATP using crude lysate.

**Supplementary Data 9.** Proteome solubility upon ATP depletion.

**Supplementary Data 10.** Codon optimized gene of BANF1.
