# Supplementary material for: Proteome-wide solubility and thermal stability profiling reveals distinct regulatory roles for ATP
Source: Nat Commun. 2019 Mar 11;10:1155. doi: 10.1038/s41467-019-09107-y (PMC6411743; doi:10.1038/s41467-019-09107-y)
Supplement: Supplementary file 14 — Reporting Summary [file 41467_2019_9107_MOESM14_ESM.pdf]

nature research

Corresponding author(s): Mikhail Savitski, Marcus Bantscheff

Last updated by author(s): Feb 11, 2019

Reporting Summary

Nature Research wishes to improve the reproducibility of the work that we publish. This form provides structure for consistency and transparency in reporting. For further information on Nature Research policies, see Authors & Referees and the Editorial Policy Checklist.

Statistics

For all statistical analyses, confirm that the following items are present in the figure legend, table legend, main text, or Methods section.

n/a	Confirmed	
☐	☒	The exact sample size (n) for each experimental group/condition, given as a discrete number and unit of measurement
☐	☒	A statement on whether measurements were taken from distinct samples or whether the same sample was measured repeatedly
☐	☒	The statistical test(s) used AND whether they are one- or two-sided<br>*Only common tests should be described solely by name; describe more complex techniques in the Methods section.*
☒	☐	A description of all covariates tested
☒	☐	A description of any assumptions or corrections, such as tests of normality and adjustment for multiple comparisons
☒	☐	A full description of the statistical parameters including central tendency (e.g. means) or other basic estimates (e.g. regression coefficient) AND variation (e.g. standard deviation) or associated estimates of uncertainty (e.g. confidence intervals)
☐	☒	For null hypothesis testing, the test statistic (e.g. F, t, r) with confidence intervals, effect sizes, degrees of freedom and P value noted<br>*Give P values as exact values whenever suitable.*
☒	☐	For Bayesian analysis, information on the choice of priors and Markov chain Monte Carlo settings
☒	☐	For hierarchical and complex designs, identification of the appropriate level for tests and full reporting of outcomes
☒	☐	Estimates of effect sizes (e.g. Cohen's d, Pearson's r), indicating how they were calculated

Our web collection on statistics for biologists contains articles on many of the points above.

Software and code

Policy information about availability of computer code

Data collection	Mass spectrometry measurements have been performed on Q Exactive Orbitrap or Orbitrap Fusion Lumos mass spectrometers (Thermo Fischer Scientific) and commercial operating software (Xcalibur) from Thermo Fischer Scientific was used
Data analysis	Mascot (Matrix Science) was used for peptide searching, isobarQuant (https://github.com/protcode/isob/archive/1.1.0.zip) was used to quantify peptide and protein abundances, R 3.4.0 was used for custom data analysis and implementation of statistical methods used (https://git.embl.de/kurzawa/sw2dTPP)

For manuscripts utilizing custom algorithms or software that are central to the research but not yet described in published literature, software must be made available to editors/reviewers. We strongly encourage code deposition in a community repository (e.g. GitHub). See the Nature Research guidelines for submitting code & software for further information.

Data

Policy information about availability of data

All manuscripts must include a data availability statement. This statement should provide the following information, where applicable:

- Accession codes, unique identifiers, or web links for publicly available datasets
- A list of figures that have associated raw data
- A description of any restrictions on data availability

All raw mass spectrometry data and respective Mascot search files have been deposited on PRIDE. Accession numbers are: PXD012423 (TPP datasets), PXD012356 (SPP datasets) and PXD012745 (Mg effect SPP dataset).

Field-specific reporting

Please select the one below that is the best fit for your research. If you are not sure, read the appropriate sections before making your selection.

☒ Life sciences ☐ Behavioural & social sciences ☐ Ecological, evolutionary & environmental sciences

For a reference copy of the document with all sections, see nature.com/documents/nr-reporting-summary-flat.pdf

Life sciences study design

All studies must disclose on these points even when the disclosure is negative.

Sample size	Biological triplicates have been used in the study to gain appropriate power for discovery of true positive events
Data exclusions	For the 3rd replicate of the GTP 2D-TPP experiment (Table S3) data corresponding to temperatures 50.4 and 51.9 C were excluded due to poor TMT labeling efficiency
Replication	Conclusions were drawn from reproducible effects in triplicates
Randomization	This is not relevant to our study, as the study utilizes biological triplicates of lysates form a single cell line with no covariates such as e.g. different patients.
Blinding	This is not relevant to our study, since data was analyzed using unbiased statistics and no covariates exist that could be analyzed with a certain bias.

Reporting for specific materials, systems and methods

We require information from authors about some types of materials, experimental systems and methods used in many studies. Here, indicate whether each material, system or method listed is relevant to your study. If you are not sure if a list item applies to your research, read the appropriate section before selecting a response.

Materials & experimental systems

n/a	Involved in the study	
☒	☐	Antibodies
☐	☒	Eukaryotic cell lines
☒	☐	Palaeontology
☒	☐	Animals and other organisms
☒	☐	Human research participants
☒	☐	Clinical data

Methods

n/a	Involved in the study	
☒	☐	ChIP-seq
☒	☐	Flow cytometry
☒	☐	MRI-based neuroimaging

Eukaryotic cell lines

Policy information about cell lines

Cell line source(s)	Jurkat E6.1 from ATCC
Authentication	authenticated using a Promega kit
Mycoplasma contamination	confirmed negative in check for mycoplasma contamination
Commonly misidentified lines (See ICLAC register)	To the best of our knowledge no misidentified cell lines have been used in this study
